# The Role of Cardiac Troponin and Other Emerging Biomarkers Among Athletes and Beyond: Underlying Mechanisms, Differential Diagnosis, and Guide for Interpretation

**DOI:** 10.3390/biom14121630

**Published:** 2024-12-19

**Authors:** Mihail Celeski, Andrea Segreti, Filippo Crisci, Riccardo Cricco, Mariagrazia Piscione, Giuseppe Di Gioia, Annunziata Nusca, Chiara Fossati, Fabio Pigozzi, Gian Paolo Ussia, Ross John Solaro, Francesco Grigioni

**Affiliations:** 1 Fondazione Policlinico Universitario Campus Bio-Medico, Via Alvaro del Portillo, 200, 00128 Roma, Italyriccardo.cricco@unicampus.it (R.C.);; 2 Unit of Cardiovascular Sciences, Department of Medicine and Surgery, Università Campus Bio-Medico di Roma, Via Alvaro del Portillo, 21, 00128 Roma, Italy; 3 Department of Movement, Human and Health Sciences, University of Rome “Foro Italico”, Piazza Lauro de Bosis 6, 00135 Roma, Italy; 4 Institute of Sports Medicine and Science, Italian National Olympic Committee, Largo Piero Gabrielli 1, 00197 Roma, Italy; 5 Department of Physiology and Biophysics and Center for Cardiovascular Research, College of Medicine, University of Illinois at Chicago, Chicago, IL 60612, USA; solarorj@uic.edu

**Keywords:** cardiac injury, cardiac troponin, exosomes, cytokines, heart failure, biomarkers, athletes

## Abstract

Cardiovascular (CV) disease remains the leading cause of morbidity and mortality worldwide, highlighting the necessity of understanding its underlying molecular and pathophysiological pathways. Conversely, physical activity (PA) and exercise are key strategies in reducing CV event risks. Detecting latent CV conditions in apparently healthy individuals, such as athletes, presents a unique challenge. The early identification and treatment of CV disorders are vital for long-term health and patient survival. Cardiac troponin is currently the most commonly used biomarker for assessing CV changes in both athletes and the general population. However, there remains considerable debate surrounding the mechanisms underlying exercise-induced troponin elevations and its release in non-ischemic contexts. Thus, there is a pressing need to identify and implement more sensitive and specific biomarkers for CV disorders in clinical practice. Indeed, research continues to explore reliable biomarkers for evaluating the health of athletes and the effectiveness of physical exercise. It is essential to analyze current evidence on troponin release in non-ischemic conditions, post-strenuous exercise, and the complex biological pathways that influence its detection. Furthermore, this study summarizes current research on cytokines and exosomes, including their physiological roles and their relevance in various CV conditions, especially in athletes. In addition, this paper gives special attention to underlying mechanisms, potential biomarkers, and future perspectives.

## 1. Introduction

Although therapeutic advancements have significantly decreased mortality from cardiovascular (CV) diseases in recent decades, the biological mechanisms underlying these illnesses remain less understood. CV events are common in the general population, with unique challenges in managing CV disorders among specific groups, such as athletes. Professional athletes who undergo prolonged physical training experience various structural and biochemical adaptations in their organ systems and body tissues [1]. However, the high costs and potential health risks associated with CV disease (CVD) management have driven the search for innovative strategies for diagnosis and treatment.

Cardiac troponin (cTn) is the most extensively studied biomarker in CV disorders, primarily due to its strong association with myocardial injury [2]. Indeed, it is the cornerstone in myocardial ischemia settings. However, myocardial ischemia is only one cause in the spectrum of possible circumstances contributing to cTn increase. Indeed, non-ischemic causes of troponin elevation must be considered often, particularly when using high-sensitivity cTn (hs-cTn) tests. These tests enable the detection of very low levels of myocardial injury, offering a more precise assessment of exercise-induced cTn release [2].

Indeed, hs-cTn assays enable the detection of cardiomyocyte damage in the acute or stable phase of established cardiac disease or even to identify in the general population the patients with either silent or clinically underestimated cardiac disease and who are therefore at a high risk of death. A special subgroup is athletes as sports-related sudden death in the general population is considerably more common than previously suspected. A recent study showed that the annual burden of sports-related sudden deaths was 4.6 per million of the population, with young competitive athletes accounting for 6% of deaths [3]. Therefore, cardiac troponin and new potential biomarkers may have significant implications in discovering latent underlying cardiac conditions or physical activity-related changes.

**Figure 1:**
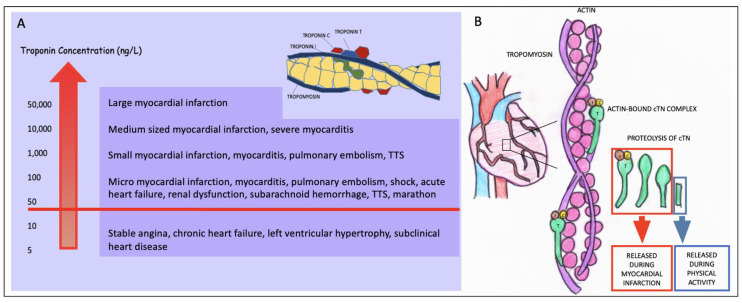
Cardiac troponin structure, range of diagnoses based on high-sensitivity cardiac troponin I concentrations (**A**) and troponin fragments release (**B**). Cardiac troponin can be released into smaller fragments during physical activity. After acute myocardial infarction, cardiac troponin complexes, intact cTn molecules, and cTn fragments (bigger and smaller in dimension) are found in circulation. After exercise, only small (15–20 kDa) cTn fragments can be isolated (**B**). TTS, Takotsubo syndrome.

Cardiac troponin is a complex molecular structure. It is a heterotrimer anchored on the thin filament that operates allosterically to control the intracellular Ca^2+^-dependent interaction of actin and myosin filaments. Different cardiac, ischemic and non-ischemic, and non-cardiac conditions contribute differently to cardiac troponin elevation (Figure 1A). However, troponin structure is more complex than it was previously thought. Since many studies have shown the existence of several circulating cTnI and cTnT forms in the blood, it was demonstrated that different cTn forms could help to distinguish acute myocardial infarction from other illnesses. Moreover, cTnI and cTnT, if related to an MI, are primarily found in a binary complex or as fragments, as described lately in [4] with larger or smaller sizes, while, after physical activity, smaller fragments (15–20 kDa) can be isolated, as demonstrated in Figure 1B.

Nevertheless, clinicians frequently face a diagnostic challenge in understanding the etiology and pathophysiology of troponin release in non-ischemic conditions. Indeed, the complexity of CVD and the limited mechanistic understanding of their pathophysiology have slowed the identification of new diagnostic and therapeutic targets. Developing new biomarkers is crucial to further reducing morbidity and mortality rates. Biomarkers—characteristics that can be objectively measured and assessed as indicators of normal or pathogenic biological processes, or pharmacologic responses—are essential for evaluating and managing CV risk [5].

Inflammation plays a central role in numerous physiological and pathological CV conditions, including exercise-induced CV adaptations. Cytokines, as signaling molecules that regulate immune responses and inflammation, also influence the body’s adaptation to physical activity. Recent studies have explored the role of cytokines in these contexts, suggesting that circulating chemokines and cytokines could serve as indicators of cardiorespiratory fitness and CV stress [6,7]. Additionally, growing evidence has highlighted the role of extracellular vesicles (EVs) in intercellular communication through the transport of bioactive molecules. Research into EVs, particularly exosomes, has advanced the understanding of their biogenesis, molecular composition, and roles in both physiological and pathological processes thereby uncovering potential diagnostic and therapeutic applications [8,9].

This review aims to provide an overview of the mechanisms behind troponin release in non-ischemic contexts, particularly following strenuous exercise, and the complexities of its detection. It also examines emerging biomarkers such as cytokines and exosomes, their physiological roles, and their implications in diverse CVD. Emphasis is placed on underlying mechanisms, their potential as biomarkers, and future research directions. Additionally, we discuss their utility in evaluating training effectiveness, exercise-induced changes, and the physiological response to physical activity.

## 2. Cardiac Troponin

When acute ischemia or other factors damage cardiac myocytes, cTn—sensitive and specific biomarkers used in the diagnosis of myocardial infarction—are released into the bloodstream. These biomarkers provide a foundation for prognosis, diagnosis, risk assessment, and the selection of revascularization and antithrombotic treatments. However, an elevation in troponin levels indicates cardiac damage rather than specifying its underlying cause. Indeed, elevated troponin levels can result from a variety of clinical situations beyond myocardial infarction [2]. In healthy individuals without myocardial injury, cTn levels typically remain within normal ranges. High-sensitivity (hs)-cTn assays however enable the detection of minor cardiomyocyte damage, potentially identifying individuals in the general population with silent or clinically unrecognized cardiac disease and, consequently, an elevated risk of mortality [10].

**Figure 2:**
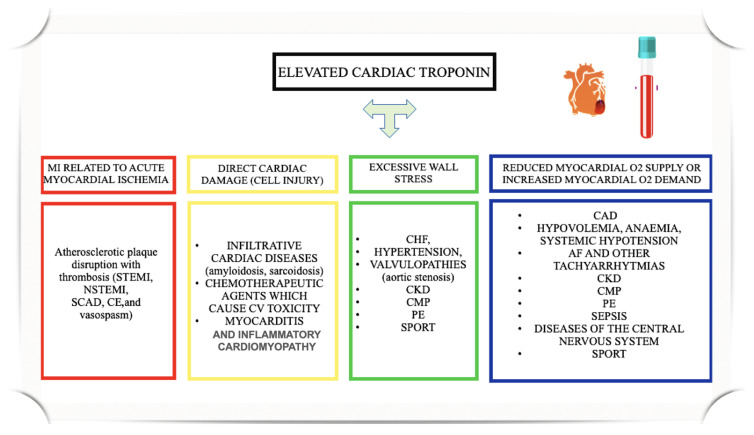
Mechanisms explaining elevated cardiac troponin in acute and chronic diseases. AF, atrial fibrillation; CE, coronary embolism; CV, cardiovascular; CHF, chronic heart failure; CKD, chronic kidney disease; CMP, cardiomyopathies; CAD, coronary artery disease; CAD, coronary artery disease; CHF, chronic heart failure; CKD, chronic kidney disease; MI, myocardial infarction; NSTEMI, non-ST elevation myocardial infarction; PE, pulmonary embolism; STEMI, myocardial infarction with ST elevation; SCAD, spontaneous coronary artery dissection.

As previously mentioned, among ostensibly healthy individuals, athletes constitute a unique subgroup. Strenuous physical exercise can elevate cTn levels, and recent studies have challenged the assumption that this increase is benign in athletes, suggesting that it may have both clinical and prognostic significance in certain contexts [11]. Nonetheless, cTn elevation can also occur in a variety of acute and chronic conditions, as illustrated in Figure 2.

### 2.1. Cardiac Troponin Increase in Non-Ischemic Conditions

Various non-ischemic cardiac and non-cardiac conditions can cause increases in cTn levels. Among the most common are heart failure (HF), cardiomyopathies, pulmonary embolism, sepsis, chronic renal failure, and stroke.

In the context of chronic HF, cardiomyocyte stretching occurs as part of myocardial remodeling [12]. In response to the reduced cardiac output associated with myocardial enlargement, the heart produces atrial natriuretic peptide (ANP) and B-type natriuretic peptide (BNP) from the atrium and ventricles, respectively. Alongside these hormones, modern assays reveal detectable cTn in 1% to 5% of asymptomatic HF patients. In contrast, most studies show that 50–80% of asymptomatic individuals have troponin I (TnI) levels above the detection limit, sometimes exceeding the 99th percentile [12]. However, the precise mechanism underlying elevated plasma cTn levels in chronic HF patients remains unclear. One hypothesis suggests that elevated cTn may result from the gradual release of cTn from the myocardium due to ongoing cardiomyocyte death, as observed in animal models with post-myocardial infarction left ventricular (LV) dysfunction [13]. Alternatively, this increase might stem from cytoplasmic vesicles (blebs) releasing cellular material into the bloodstream due to a mismatch between oxygen demand and supply, especially in subendocardial layers [14]. Another theory posits that aberrant calcium metabolism in HF activates intracellular proteolytic enzymes, breaking down cTn and releasing fragments detectable in cTn immunoassays [15]. Despite ongoing debates regarding cTn’s role in HF, cTnI assessment may be essential for accurate risk stratification [16]. Furthermore, troponin isoforms undergo important developmental and post-translational modifications, underscoring cTn’s complex role in regulating cardiac function, especially in HF and hypertrophic cardiomyopathy [17]. In HF patients, elevated cTn may indicate either myocardial injury from acute decompensated HF or an acute coronary syndrome (ACS), representing a critical challenge for clinicians. Since ischemic heart disease is a prevalent cause of HF, coronary angiography can sometimes clarify the clinical picture, and using additional biomarkers with cTn may enhance the accuracy of distinguishing acute decompensated HF from ACS.

Other cardiac conditions associated with cTn elevation include cardiomyopathies. cTn measurements in plasma or serum are valuable for staging CVD, stratifying treatment, and predicting prognosis. In hypertrophic cardiomyopathy (HCM), hs-cTn elevation is linked to CV events, HF, and mortality, warranting its inclusion in the diagnostic workup [18]. cTn levels in HCM are generally higher than in hypertension but lower than in infiltrative cardiomyopathies. Notably, in amyloidosis, a non-sarcomeric HCM with extracellular amyloid fibril deposits, high cTn levels predict poor prognosis [19]. Elevated cTn, observed in 4–66% of HCM cases, often exceeds the recommended 99th percentile cut-off threshold [19]. Some studies suggest that LV wall thickness and diastolic dysfunction contribute to cTn release [20], with associations found between the left atrial diameter and cTn elevation. In dilated cardiomyopathy (DCM), where significant coronary artery disease is absent, cTn levels are lower than in ischemic conditions. Ischemic LV dysfunction shows higher cTn levels than DCM, although cTn remains elevated over 3 months [21]. Nonetheless, cTn assessment has potential in DCM, where elevated levels correlate with poorer outcomes [22]. In arrhythmogenic cardiomyopathy, the correlation between cTn and disease progression is weaker, with cTn elevation potentially linked to ventricular arrhythmias [23]. As cTn gene mutations contribute to cardiomyopathy, further research is needed to clarify the role of genotype in cTn blood levels [24].

Stress-induced cardiomyopathy, or Takotsubo syndrome (TTS), also leads to elevated cTn [25,26]. In TTS, catecholamine release, such as noradrenaline and adrenaline, may lead to myocardial injury, though the permanence of this damage is not fully understood [27]. Evidence suggests that TTS’s acute phase involves inflammation, metabolic, and microvascular changes, which may contribute to cTn elevation [28].

Myocarditis and inflammatory cardiomyopathy are other important causes of cTn elevation. Myocarditis combined with cardiac failure and ventricular remodeling is known as inflammatory cardiomyopathy. Although cardiac troponins are more sensitive to myocyte damage than creatine kinase levels in patients with clinically suspected myocarditis, they are not specific and do not rule out myocarditis when they are normal [29]. Indeed, in myocarditis, autoantibody formation against cTn could exacerbate cardiac damage. Moreover, a potentially fatal arrhythmia may be preceded by fluctuations in cardiac troponin concentrations [29]. On the other hand, when a dilated left ventricle is present and there is a slight increase in troponin levels in plasma that is out of proportion to the degree of LVEF impairment, this suggests inflammatory cardiomyopathy rather than acute myocarditis [30].

Pulmonary embolism (PE) can also elevate cTn, often due to right ventricular (RV) dilation or cardiogenic shock. Right ventricular ischemia and injury, arising from increased oxygen demand and intramural pressure, lead to endothelial mediator release [31]. The cTn increase in PE is generally moderate, reflecting cytosolic myocyte damage. Studies show that cTn can clear within 24 h due to lower RV cTnT and cTnI tissue content [32]. Still, cTn assessment is crucial in stratifying and managing PE patients.

In non-cardiac conditions, cTn can elevate in sepsis, likely due to increased membrane permeability and troponin release [33]. Myocyte injury in sepsis appears transient, as reversible myocardial depression occurs upon recovery [34]. COVID-19 and other viral infections can also elevate cTn [35]. A troponin increase in COVID-19 has clinical and prognostic implications. Indeed, in a recent study evaluating troponin release among COVID-19 patients, cTn elevation was an independent predictor of 30-day mortality, suggesting that the early measurement of cardiac troponin may be useful for risk stratification in COVID-19 [36]. On the other hand, raised troponin I levels in COVID-19 patients could indicate direct viral myocarditis, coronary vascular ischemia, or an exacerbated inflammatory response. Therefore, assessing troponin I and other cardiac biomarker levels may aid in the triage of severe COVID-19 patients at risk for major cardiovascular events and enable timely management in order to optimize prognosis and reduce risk for [37].

Moreover, in chronic kidney disease (CKD), elevated cTn levels correlate with poor prognosis [38]. Possible explanations for cTn elevation in CKD include unbound cTn fraction release, reduced clearance, or the formation of cTn immunoactive fragments detectable by immunoassays [38,39].

Cerebrovascular events, including subarachnoid hemorrhage (SAH) and ischemic stroke, may also elevate cTn. In hemorrhagic stroke, catecholamine release induces contraction band necrosis, while ischemic stroke studies show inconsistent cTn results [40,41].

Although the mechanisms of cTn release in non-ischemic conditions are still under investigation, its assessment is essential in clinical practice. After excluding ischemic causes, further laboratory and imaging studies are needed to identify the etiology of cTn elevation.

### 2.2. Cardiac Troponin Elevation in Athletes and Underlying Mechanisms

It is now well established that cTn levels can increase following sustained, intense physical activity (PA). Numerous studies have shown that the kinetics of cTn after PA do not necessarily indicate myocardial damage, as these increases are typically transient, with levels often returning to normal within 48 h [42]. Research on the clinical significance and predictive value of exercise-induced cTn increases however remains limited. For this reason, elevated cTn levels in athletes have traditionally been regarded as a normal physiological response to exercise.

Recent studies however suggest that exercise-induced elevations in cTn may not always be benign; they may, in certain cases, indicate a higher risk of future CVD and mortality. Aengervan et al., for example, reported that individuals with post-exercise cTnI levels above the 99th percentile (>0.040 μg/L) experienced significantly higher rates of a composite endpoint—including all-cause mortality and major adverse CV events ((MACE) encompassing myocardial infarction, stroke, HF, revascularization, or sudden cardiac arrest)—compared to the controls with cTnI levels ≤ 0.040 μg/L (27% vs. 7%, log-rank *p* < 0.001) [43].

Elevated cTn levels at rest and during exercise have also been linked to CV risk factors and coronary artery disease (CAD), with particularly high levels observed in athletes engaged in prolonged high-intensity exercise [44]. While many studies have confirmed that endurance exercise promotes CV health and extends survival, evidence also shows that long-term high-intensity exercise can lead to adverse changes, such as increased platelet aggregation, left ventricular wall motion abnormalities, and, in rare cases, ischemic events [44].

**Figure 3:**
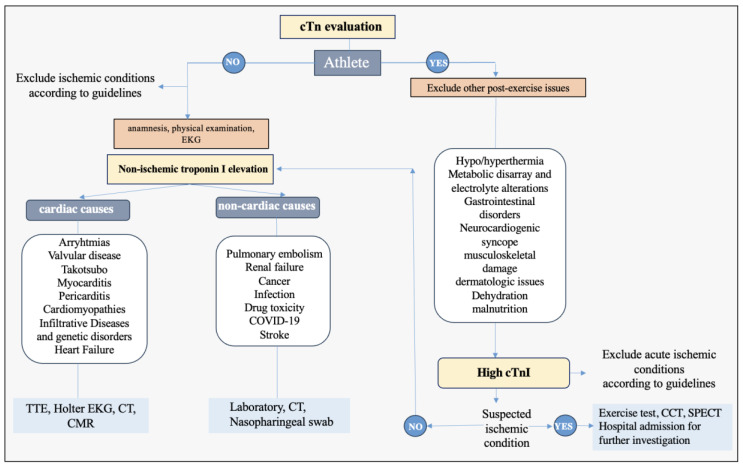
Algorithm for interpreting non-ischemic cardiac troponin (cTn) release in both athletic and non-athletic populations. EKG, electrocardiography; TTE, trans-thoracic echocardiography; CT, computed tomography; CCT, cardiac computer tomography; CMR, cardiac magnetic resonance; cTn, cardiac troponin; SPECT, Single Photon Emission Computed Tomography.

The release of cardiac biomarkers in athletes is influenced by several factors, including anatomical, demographic, CV, and technical aspects [11]. Additionally, the extent of cTn elevation may vary based on the type of workout—whether dynamic, static, or a combination of both. Recent research further suggests that troponin elevation in athletes may have clinical and prognostic significance in some cases, complicating the interpretation of cTn test results, particularly in athletes displaying symptoms suggestive of cardiac conditions [11]. The primary non-ischemic and athlete-specific conditions that contribute to cTn release are illustrated in Figure 3.

The exact mechanisms behind the release of cTn from cardiomyocytes into the circulation remain uncertain. If we consider elevated cTn a marker of irreparable damage, then its release might indicate necrosis. However, four other plausible mechanisms were proposed [45].

Firstly, PA can disrupt the cardiomyocyte cell membrane through contraction, beta-adrenergic stimulation, stretching, or transient ischemia, allowing intracellular substances, including cTn, to pass into the bloodstream [46]. cTn fragments may enter circulation through exocytosis, microvesicles, small cellular injuries, or passive diffusion, especially as membrane permeability increases during PA [46]. In this scenario, cardiomyocyte injury does not necessarily involve cell death or necrosis and may be reversible through reoxygenation. The heart’s increased blood flow demand during PA, driven by elevated heart rate and stroke volume, may lead to size alterations in cTn fragments, allowing cTnI and cTnT to pass through the membrane [47]. Studies by Vroemen et al. found only small cTnT fragments in marathon runners’ blood. Post-race hs-cTnT levels exceeded the diagnostic threshold for acute myocardial infarction (AMI), with fragment sizes comparable to those in end-stage renal disease patients, suggesting a similar release mechanism [45].

Secondly, cTn release may occur through apoptosis, a process associated with rapid cell turnover [48]. However, since apoptotic cells are typically broken down and absorbed by other cells, it is less likely that their contents would be released extracellularly. Exercise-induced catecholaminergic stress transiently increases myocardial apoptosis by approximately 150%. Excessive cardiac workload, especially in untrained individuals, correlates with increased apoptosis and cTn release due to elevated left ventricular preload [49,50]. Additionally, volume expansion and subendocardial ischemia can activate necroptosis pathways, leading to cTn release [51].

Thirdly, the most plausible mechanism remains myocardial ischemia, which causes a metabolic shift from aerobic to anaerobic pathways, resulting in the release of intracellular proteins into circulation [52]. While CMR often fails to detect edema or scarring after intense PA, suggesting minimal necrosis, it may lack the sensitivity needed to detect small-scale necrosis [53].

Lastly, an alternative non-cardiac explanation involves hemoconcentration, dehydration, and impaired renal function due to rhabdomyolysis from extreme exercise. In addition, cTn assays might cross-react with skeletal troponin or reflect skeletal muscle injury. While skeletal muscle damage may contribute to exercise-induced cTnT increases, it likely has a limited effect on cTnI levels [54].

Given these ongoing uncertainties, athletes experiencing exercise-induced cTn elevations should undergo a comprehensive diagnostic evaluation (Figure 3). Although endurance athletes generally have a low risk of atherosclerotic CAD, there remains a minor but significant risk that can be underestimated [55]. Athletes presenting to the emergency department with elevated cTn post-exercise should receive appropriate evaluation, including assessment to exclude non-cardiac conditions, a 12-lead ECG, serial cTn measurements, and noninvasive or invasive risk stratification based on the clinical context [11].

Further techniques may help clarify these mechanisms. Gel filtration chromatography could differentiate cTn fragments by molecular size, aiding in distinguishing normal from pathological cTn elevations [56,57]. Examining different cTn fragments is a promising method for this differentiation because post-myocardial infarction and post-exercise may have different cTn fragment compositions. Indeed, following their release from cardiac cells, cTnI and cTnT are prone to proteolysis [58]. Enzymes cleave the N- and C-terminal parts of cTnT, producing smaller primary and secondary circulating fragments. Because MI patients have a release of cTn T–I–C complexes, whole cTnT and cTnI, and fragments of T and I, this may have clinical significance. It is possible to make distinctions between principal 29 kDa cTnT fragments and secondary 15–20 kDa cTnT fragments. Only tiny secondary cTnT fragments, ranging in size from 15 to 20 kDa, are detected after a marathon run. Current cTn tests employ antibodies against epitopes in the core regions of cTnI and cTnT, which have greater stability [58]. Another promising approach involves using cardiac organoids derived from human stem cells for in vitro studies. These engineered tissues, containing cardiomyocytes, could undergo simulated exercise and subsequent cTn release analysis, with advanced techniques like high-resolution microscopy and biochemical staining applied to assess damage [59]. Further studies exploring the complex biology surrounding cTn and its microenvironment may ultimately resolve some of these uncertainties.

### 2.3. The Complex Biology Behind Cardiac Troponin Release

The release of cTn into the bloodstream involves a complex interplay of mechanical forces and cytoskeletal dynamics. Understanding these mechanisms is critical for accurately interpreting serum cTn levels in diagnosing and prognosing cardiac conditions, especially in contexts where cardiac myocyte integrity is impacted by stress [53]. During AMI, cTn is primarily released due to cell rupture and bleb formation. However, cTn release mechanisms are intricate, involving mechanical forces acting on the sarcomeres of cardiac myocytes within a mechano-transduction network. This network includes the extracellular matrix (ECM), integrins, Z-disk and M-band proteins, costameres, and microtubules, all essential for maintaining membrane integrity and influencing cTn release during cardiac stress [60].

Research suggests that cTn can be released without complete cellular necrosis, as ischemic changes in the cytoskeletal network can cause surface blebs. These blebs do not necessarily lead to cell rupture but may result from alterations in membrane mechanics, influenced by calpain and integrin activation. The Z-band and cytoskeletal attachments are crucial for membrane stability, and their breakdown can lead to membrane fragility and subsequent cTn release [60]. Other studies have shown that the loss of α-actinin—a key component of the Z-disk and cytoskeletal connections in the sarcomere—indicates the presence of cTnI breakdown products under ischemic stress in perfused hearts [61].

The relationship between external forces (outside-in stresses) and cTn release was also established, involving complex interactions among microtubules, proteases, cytokines, sub-sarcolemma elements, and the cytoskeleton. Microtubules play a significant role in maintaining membrane stability and in the potential release of cTn into the bloodstream [62]. Elevated end-diastolic stress can trigger the degradation and release of cTnI, and oxidative stress has been shown to alter myofilament calcium sensitivity, further contributing to this process [60].

Internal (inside-out) stresses are also relevant, particularly in HCM, where sarcomere and cytoskeletal mutations disrupt normal function. These stresses can trigger biochemical changes that elevate cTn levels, often reflecting underlying cardiac dysfunction. In HCM, increased cTnI or cTnT levels have been linked to subclinical disorders, sometimes preceding significant structural abnormalities. Possible mechanisms include microvascular alterations, imbalances between energy supply and demand, myocardial wall thickening, and myocyte dysfunction, all contributing to membrane instability. Such processes may involve sarcomere and cytoskeletal strain, programmed necrosis, sarcolemma instability, and even exosome release [60].

Genome-wide association studies (GWAS) involving biomarkers have emerged as valuable tools for understanding elevated serum cTn in apparently healthy individuals, such as athletes, who engage in intense or prolonged exercise [60]. Thin filament activation, responsive to Ca^2^⁺, may underlie the intrinsic length–tension properties of cardiac myocytes and the Frank–Starling mechanism. Importantly, the human genome contains single-copy genes encoding cTnC, cTnI, and cTnT, which are dynamically regulated and cardiac-specific [63].

The immune system also plays a role in cardiac damage and cTn release. Cardiac immune cells include recruited immune cells, natural killer (NK) cells, cardiac tissue resident macrophages (cTMs), and resident immune cells. These cells maintain cardiac function by phagocytosing bacteria and necrotic cells, regulating proliferation, inflammation, and fibrosis, and contributing to extracellular matrix and collagen production. Despite advancements in molecular and cellular therapies, gaps remain in understanding the immune microenvironment’s role in cardiac injury repair [64].

Additional studies across the general population and diverse cardiac disorders are needed to elucidate the role of the microenvironment in cTn release and to uncover mechanisms that remain poorly understood.

## 3. Exosomes

### 3.1. Biogenesis and Function of Exosomes

Exosomes are a type of extracellular vesicle with a spherical shape, bounded by a lipid membrane, averaging less than 150 nm in diameter. They are involved in cell–cell communication, a fundamental process in living organisms that regulates metabolism, promotes adaptation, and ensures survival [65,66]. Exosomes are released through the fusion of the multivesicular body (MVB) with the plasma membrane. This process, discovered over 30 years ago, has sparked growing interest in exploring the role of exosomes [65]. Biogenesis begins with the activation of a receptor on the plasma membrane [67], which mediates endocytosis of the ligand–receptor complex with membrane components and encapsulates proteins and genetic material in the cytoplasm, forming early endosomes [68,69]. The early endosome matures into a late endosome, containing the MVBs [70]. Once the late endosome is formed, it either fuses with lysosomes for content degradation or with the plasma membrane to release its contents as exosomes. Mechanisms that favor exosome secretion over lysosomal degradation, involving proteins such as the Endosomal Sorting Complex Required for Transport (ESCRT) complex, Rab proteins, and tetraspanin 6, remain only partially understood [71,72]. Upon release, exosomes interact with recipient cells through three main mechanisms: phagocytosis, micropinocytosis, and endocytosis [73].

Exosomal membrane proteins such as Intercellular Adhesion Molecule 1 (ICAM-1) can engage receptors on target cells, triggering intracellular signaling, or interact with receptors on the exosomal membrane, such as tumor necrosis factor (TNF) receptor 1 (TNFR1) or cluster of differentiation (CD) 46, for specific functions [74,75,76].

Exosomes contain diverse proteins, lipids, and nucleic acids, with composition influenced by cellular conditions and treatments, potentially leading to heterogeneity [70]. Once released, exosomes perform physiological roles like immune response induction, stem cell maintenance, and tissue repair [77,78,79], but they can also contribute to pathological processes, such as neurodegenerative diseases [80,81] or CVD like peripartum and sepsis-induced cardiomyopathy, tumor thrombosis, and angiogenesis [82]. Due to their roles in pathology and physiology, exosomes have potential applications in the diagnosis, prognosis, and treatment of numerous diseases [83].

### 3.2. Role of Exosomes in Different Cardiovascular Conditions

The adverse health impact and economic burden of managing CVD have prompted research into novel therapeutic strategies for early diagnosis and treatment [84]. In recent years, exosomes have gained substantial attention in this context [85]. While cardiomyocytes comprise one-third of total heart cells, other cell types, including fibroblasts, smooth muscle cells, endothelial cells, neuronal cells, inflammatory cells, and cardiac-derived stem cells, also play vital roles [86]. The focus has recently shifted toward understanding cell-to-cell communication mechanisms among these cell types in both pathological and physiological conditions [87]. Exosomes, due to their role in the transport and exchange of signaling molecules, could be critical in regulating CVD progression [88,89], including conditions such as atherosclerosis.

Atherosclerosis, a pro-inflammatory condition involving complex vascular remodeling, is characterized by endothelial dysfunction, smooth muscle cell proliferation and migration, and inflammatory cell infiltration in response to environmental factors. In this setting, exosomes from endothelial cells, smooth muscle cells, and immune cells mediate cell communication and amplify inflammatory responses [90,91]. Depending on the cell of origin, exosomes can either promote or inhibit atherosclerosis [85]. For instance, exosomes from atherosclerotic plaques carry adhesion molecules, such as ICAM-1, which advance plaque progression through immune cell recruitment [92]. Similarly, exosomes from dendritic cells, through TNF-α and NF-kB signaling, promote adhesion molecules like VCAM-1, ICAM-1, and E-selectin thereby heightening inflammation and atherosclerosis [93].

On the other hand, exosomes secreted by proliferative vascular smooth muscle cells (VSMCs) are enriched with fetuin-A, a potent glycoprotein that inhibits calcification and aids in vascular repair processes [94]. Additionally, exosome-dependent heat shock protein 70 (HSP70) triggers monocyte activation, leading to monocyte adhesion and a pro-inflammatory response, which contributes to the onset of atherosclerosis [95]. Exosomes derived from macrophage T-HP 1 cells are enriched with miR-146a, promoting reactive oxygen species (ROS) generation and neutrophil extracellular traps (NETs) through the downregulation of superoxide dismutase 2 (SOD2) expression. Intravenous administration of miR-146a-enriched exosomes from oxLDL-treated THP-1 cells in a murine model of atherosclerosis was shown to exacerbate atherosclerosis [96]. Exosomes from mesenchymal stem cells may also play a crucial role in atherosclerosis, reducing macrophage infiltration, promoting M2 reparative polarization, and alleviating the condition. Furthermore, platelet-derived exosomes exhibit diverse physiological effects on the inflammatory process in response to various pathogenic stimuli [97].

Studies have also demonstrated that endothelial cell-derived exosomal miR-501-5p targets Smad3, promoting VSMC proliferation and migration, and contributing to in-stent restenosis. Additionally, exosomes from M2-type macrophages can upregulate activator protein-1, a transcription factor involved in VSMC proliferation, migration, and dedifferentiation, suggesting a potential therapeutic approach for vascular tissue repair and reducing in-stent restenosis incidence [98,99].

AMI is typically identified by acute myocardial injury indicated by elevated troponin levels alongside evidence of myocardial ischemia. However, exosomes may also play a significant role [2]. During AMI, exosomal cargo is modified to contain cardioprotective factors such as specific miRNAs (e.g., miRNA-214, -1, -208, -22, -133a) and HSP70 from bone marrow-derived stem cells [100]. Bo Wang et al. confirmed that circulating exosomal miR-342-3p levels are low during the acute phase of MI, which correlates with compromised myocardial function. Physiologically, miR-342-3p targets the TFEB and SOX6 genes, inhibiting autophagy and apoptosis in myocardial cells [101]. Research shows that patients with myocardial ischemia exhibit a low expression of miR-939-5p in coronary artery serum exosomes, which results in the decreased inhibition of inducible nitric oxide synthase (iNOS), thus increasing NO synthesis and enhancing vascularization [102].

Another study proposed that exosomes from peripheral blood in acute MI patients carry miR-126-3p, which targets mTORC1 and enhances hypoxia-inducible factor (HIF-1a) expression, promoting angiogenesis by upregulating vascular endothelial growth factor (VEGF), a critical mediator of vascularization [103,104]. Additionally, circulating exosomes from patients with ST-segment elevation AMI are rich in sphingolipid species, such as ceramides, dihydroceramides, and sphingomyelins, with elevated levels correlating with cTn, leukocyte count, and decreased left ventricular ejection fraction [105].

Macrophages are essential in the immune response following AMI, initially driving a pro-inflammatory response and subsequently aiding inflammation resolution and tissue repair [106]. Recent findings indicate that M1 macrophages secrete exosomes containing miR-155, which reduces endothelial cell migration and suppresses angiogenesis, thereby worsening myocardial injury. Conversely, M2 macrophage-derived exosomes containing miR-1271-5p can reduce myocardial apoptosis in acute MI and promote cardiac repair [107]. Studies confirm that miR-30a-rich exosomes released by hypoxic myocardial cells regulate autophagy in other myocardial cells by inhibiting proteins like Beclin-1 [108].

In acute MI, exosomes play a protective role by facilitating cross-talk between cardiac and body mesenchymal cells thus modifying the microenvironment for long-term re-education [109]. Circulating levels of exosomal lncRNAs were evaluated as potential biomarkers in AMI patients [110].

Exosomes also hold relevance in HF. Studies have linked systemic vascular markers such as CD14, SerpinG1, and SerpinF2 with HF progression. A recent study showed increased exosomal levels of inflammation-associated miRNAs like miR-146a and miR-486 in HF patients [111,112]. Chronic activation of the myocardial renin-angiotensin system (RAS) and subsequent increased angiotensin II levels contribute to HF pathophysiology. The treatment of cultured cardiomyocyte fibroblasts (CFs) with Ang II increases exosome release by activating Ang II receptors type 1 (AT1R) and 2 (AT2R), leading to hypertrophy through the upregulation of renin, angiotensinogen, and AT1R/AT2R while downregulating ACE2 [113]. Furthermore, the in vivo administration of exosomes derived from cardiac progenitor cells (CPCs) improved cardiac function and reduced fibrosis in an ischemia–reperfusion rat model [114].

Cardiomyopathies, a group of myocardial diseases that can lead to progressive HF, were also linked to exosomes due to their pro-inflammatory roles during disease onset [84]. However, their role in cardiomyopathies is still under investigation.

Similarly, exosomes may influence valvular disease development. During development, exosomal miRNAs are involved in regulating cardiac valve formation by modulating target genes [115]. Yang et al. reported an association between exosomal miRNAs and myxomatous mitral valve disease in a canine model. Exosomal miRNAs involved in cardiomyocyte energetics, fibrosis, and mitochondrial function, such as miR-9, miR-181c, miR-495, and miR-599, are linked to valve disorder progression and congestive HF [116]. Similarly, Carrion et al. identified a mechanistic association between lncRNA HOTAIR and calcification of VSMCs in aortic valve disease [117]. The role of exosomes in various cardiac conditions however remains an active area of investigation.

### 3.3. Role of Exosomes and Interpretation Among Athletes

Exosomes play a role in tissue repair and regeneration, making them potentially valuable in sports medicine and enhancing athletic performance [118]. Exosomes facilitate myo-trauma remediation and restoration by stimulating myogenic proliferation, catalyzing tendinous cell maturation, fostering neurite outgrowth, and promoting Schwann cell proliferation [119].

Specifically, exosomes derived from adipose-derived mesenchymal stromal cells show therapeutic potential for myogenic regeneration, while those from bone marrow stromal cells (BMSCs) enhance muscle healing by promoting M2 macrophage polarization [120,121]. Some studies indicate that exosomes from BMSCs can suppress TGFBR1 expression, which helps impede the progression of adhesive capsulitis [122,123]. Similarly, exosomes from tendon stem cells may support tendon healing by balancing the synthesis and degradation of the extracellular matrix [124]. Exosomes also show therapeutic promise in tendon–bone healing, particularly in anterior cruciate ligament reconstruction. Studies demonstrate that BMSC-derived exosomes modulate M1/M2 macrophage polarization thereby facilitating tendon–bone healing [125]. Additionally, exosomes have potential in arthritis treatment, as they can alleviate cartilage damage, inhibit bone overgrowth, and modulate immune responses by reducing T lymphocyte proliferation and other inflammatory effects [126].

Recent studies highlight the significant role of exosomes in metabolic regulation during physical activity, particularly endurance exercise [127]. Research indicates that endurance exercise triggers the release of exosomes, rich in peptides and nucleic acids, from skeletal muscle and other tissues [128]. Moreover, studies suggest that circulating exosome concentration rises with increased exercise intensity, underscoring a critical role in metabolic regulation during physical activity [128,129]. In terms of anti-fatigue properties, exosomes could improve cellular energy metabolism and enhance resistance to damage. Their mechanisms in cellular bioenergetics include enhancing mitochondrial efficiency, amplifying adenosine triphosphate (ATP) synthesis, and optimizing oxidative phosphorylation [130,131]. In addition, exosomes may play an antioxidative role by sequestering and neutralizing reactive oxygen species [132].

Exosomes are also crucial in myocyte repair, which is highly relevant for athletes undergoing rigorous training [118]. Research has confirmed that exosomes can carry growth factors, microRNAs, and other bioactive compounds to myocytes following trauma, aiding in tissue restoration and potentially enhancing athletic stamina [133,134]. As previously mentioned, exosomes can modulate immune responses and may help reduce post-exercise inflammatory reactions [135]. However, their full clinical potential in athlete management and performance enhancement remains unexplored.

## 4. Cytokines

### 4.1. Cytokines–From their Definition to their Role in Cardiovascular Pathophysiology and Exercise

Cytokines are small signaling molecules with endocrine, autocrine, and paracrine effects involved in immunomodulation. Primarily released by immune cells—including macrophages, dendritic cells, neutrophils, natural killer cells, monocytes, eosinophils, basophils, and lymphocytes—cytokines communicate with other cells to orchestrate immune responses during inflammation [136]. There are various types of cytokines, such as interleukins (ILs), TNF, interferons (IFNs), chemokines, colony-stimulating factors (CSFs), and growth factors. The balance between pro-inflammatory and anti-inflammatory cytokines is essential for maintaining homeostasis; the disruption of this balance can lead to immune pathologies [137]. Inflammation is closely related to reactive oxygen species (ROS) production, which can lead to oxidative stress, cell damage, and mutations [138]. Certain cytokines may play crucial roles in exercise and CV health, especially in relation to atherosclerosis [139].

Although descriptive information was published summarizing exercise-induced inflammation and cytokine expression, a detailed understanding of signaling arising from levels of exercise and altered function in the cardiac microenvironment is lacking. An emerging mechanism in this signaling is Hippo signaling, which is an evolutionally conserved and overarching complex path of sensing the mechanical and metabolic state of tissues by controlling ON and OFF states controlling the expression of genes via two major downstream effectors, Yes-associated protein (YAP) and its homolog transcriptional coactivator with PDZ-binding motif (TAZ) into the nuclear compartment [140]. Hippo signaling was identified as an element in myocyte homeostasis with aging and exercise [141,142]. Moreover, Hippo signaling was reported to be significantly involved in modulating pro-inflammatory cytokine expression in animal models stressed with isoproterenol treatment as a surrogate for MI [143]. Dysfunction in Hippo signaling alters gene expression, promoting myocardial injury by promoting apoptosis, mitochondrial fission, oxidative stress, and calcium overload. These data indicate a need for further exploration of the role of altered Hippo signaling in exhaustive exercise, where elevated serum cytokine levels are demonstrated.

Patients with atherosclerosis typically exhibit elevated serum interleukin (IL) 6 levels, with higher levels observed in patients with unstable angina compared to those with stable angina [144,145]. Oxidized low-density lipoproteins (LDL) present in atherosclerotic plaques, which are central to plaque formation and destabilization, promote TNFα production in peripheral blood [146]. Elevated cytokine levels may also have prognostic significance. For example, serum IL-18 levels were shown to be independent risk predictors of mortality in patients with coronary atherosclerosis [147]. Additionally, the IL-1 receptor antagonist (IL-1ra) blocks IL-1α and IL-1β from binding to their receptors thereby limiting their pro-inflammatory activity. Elevated IL-1ra levels were observed in patients with unstable coronary disease who experienced a complicated hospital course [145].

Other CV diseases, including chronic coronary syndrome and HF, are associated with increased levels of pro-inflammatory cytokines such as interferon-γ, IL-1β, IL-6, and TNF-α [148]. In patients with AMI undergoing percutaneous coronary intervention (PCI), serum concentrations of IL-4 and IFN-γ were shown to predict left ventricular dysfunction development and are associated with a poor prognosis post-AMI [149]. Furthermore, IL-6 was identified as a strong predictor of future MI risk in healthy men and is also a predictor of 30-day mortality in AMI patients with cardiogenic shock [150]. Persistently elevated TNFα levels were found in patients who experienced recurrent coronary events following AMI when compared to the controls [151].

Genetic variants associated with higher plasma levels of IL-5 (a cytokine secreted by T helper 2 cells that acts on eosinophils to release proteins like eosinophil basic protein, neurotoxin, peroxidase, and leukotrienes) were also linked to an increased risk of coronary artery disease [152].

There is a strong association between inflammation and HF as well. Patients with HF have significantly higher levels of IL-6 and TNFα compared to healthy individuals [153]. Studies have shown an elevated expression of inflammatory cytokines in HF patients, including TNFα, IL-1, IL-6, IL-18, and various chemokines such as monocyte chemoattractant protein-1 (MCP-1/CCL2), IL-8/CXCL-8, CXCL-16, and CCL-21 [154,155]. Inflammation is especially relevant in the pathogenesis of HF with preserved ejection fraction (HFpEF), as patients with HFpEF show higher circulating IL-6 and IL-8 levels than those with asymptomatic hypertension [156].

Inflammatory cytokines affect myocardial tissue through various mechanisms. For example, their interactions with cardiomyocytes and fibroblasts can lead to hypertrophy and fibrosis, impairing myocardial contractile function. Cytokines also influence intracellular calcium transport and signal transduction through interactions with β-adrenergic receptors, induce apoptosis, and stimulate gene expression related to myocardial remodeling [157]. TNF-α and IFN-γ trigger monocytes and macrophages to produce IL-1 and IL-6, which act on endothelial and smooth muscle cells within the arterial wall [158].

Given the emerging evidence of their role in CV disease pathogenesis, some clinical trials have explored the use of interleukin blockers. The CANTOS study demonstrated a lower rate of recurrent CV events with the use of Canakinumab, an IL-1 blocker [159]. After AMI, excessive inflammation can lead to thrombosis and post-AMI complications. Reducing myostatin—a cytokine released by muscle cells during exercise—was shown to inhibit pathological cardiac remodeling in ischemic cardiomyocytes [160]. Myostatin is also implicated in atherosclerosis and AMI [161]. Other clinical trials focusing on immune modulation post-AMI have demonstrated reductions in inflammatory response, favorable effects on cardiac remodeling, and a decrease in HF events [162,163,164,165].

Inflammation and cytokines are also associated with arrhythmias. TNF, IL-1, IL-6, and IL-17 can influence ion currents in cardiac cells, prolonging action potential duration and increasing the risk of ventricular arrhythmias, atrial fibrillation, and conduction disturbances [166].

### 4.2. Biomarkers in Predicting Cardiovascular Diseases

Given the significant role cytokines play in CVD, they have gained interest as alternative biomarkers to classic indicators, such as cTn. Elevated levels of TNF-α, IL-6, IL-1, and adipokines like adiponectin, visfatin, and resistin are associated with higher mortality and morbidity, potentially enhancing prognostic models in AMI [167]. Studies have shown that elevated interleukin levels in plasma and serum correlate with adverse outcomes in HF patients, particularly with worsened functional class and cardiac performance. Increased plasma levels are noted in patients with higher NYHA classes and reduced left ventricular ejection fraction [154,168]. In HF patients undergoing left ventricular assist device (LVAD) implantation, a study by Diakos et al. demonstrated that a lower cardiac and systemic inflammatory burden is associated with greater cardiac improvement post-implantation. They also developed a two-cytokine predictive model using baseline IFN-γ and TNF-α levels to help identify patients more likely to experience structural and functional improvement after LVAD implantation [169]. Cytokines show promise as biomarkers for predicting prognosis and progression in CVD. However, they are less effective in diagnosing or predicting acute CVD and future coronary heart disease in asymptomatic individuals [170]).

Important immune cells and cytokines were also identified by mechanistic investigations as contributing to inflammation in myocarditis. The innate immune system contributes during the acute phase, whereas the acquired immune system does so during the chronic phase. Although cytokines have a significant role in pathogenesis, they also have clinical and prognostic effects in inflammatory cardiomyopathy and myocarditis. For instance, TGFβ, endothelin-1, connective tissue growth factor, and platelet-derived growth factor D (PDGF-D) are significant cytokines linked to the pathophysiology of Chagas disease [171]. On the other hand, male patients with acute myocarditis have worse prognosis if there are higher levels of the circulating serum soluble ST2 receptor (sST2), an IL-33 receptor that has predictive significance in chronic HF and inflammatory cardiomyopathy [171]. Moreover, IL-8, IL-1b, and IL-12 can be implemented in the risk stratification of infective myocarditis [172]. In this context, cytokines may also play a therapeutic role. In fact, a key role in the pathophysiology of cardiac inflammation is played by the pro-inflammatory cytokine IL-1. Conversely, timely and intentional pharmacologic suppression of IL-1 reduces tissue damage and uncontrollable inflammation, reducing arrhythmias and recovering cardiac function. IL-1 inhibition may therefore be a potentially good treatment choice for myocarditis due to its dual efficacy against contractile failure and cardiac inflammation [173].

However, the routine use of cytokines in everyday clinical practice is challenging for several reasons. Plasma cytokine levels vary widely across populations and are influenced by factors such as age, sex, circadian rhythm, postprandial variation, lifestyle, and recent physical activity. It is well established that cytokine levels increase with age [174], and in AMI patients, IL-6 levels are significantly higher in older individuals, indicating a worse prognosis with age [175]. Without standardized, age-adjusted normal ranges for cytokine plasma levels, interpreting these values in clinical settings is difficult. Cytokine production is also influenced by circadian rhythms, which are in turn affected by melatonin and cortisol secretion [176].

Body mass index (BMI) correlates with higher levels of C-reactive protein (CRP), and diet also plays a role: red meat consumption is associated with elevated IL-6 and IL-8 levels, whereas a Mediterranean diet shows no such associations when adjusted for BMI [177]. Physical exercise further complicates cytokine assessment, as some cytokines, known as “myokines”, are produced and released by muscle cells in response to contraction [178]. IL-6, in particular, increases after muscle contraction and is well studied as a key exercise-related cytokine. Additionally, smokers have higher circulating cytokine levels [177].

Proper blood or plasma sample collection is critical to avoid analytical biases. Some cytokines have short half-lives and require rapid processing [179]. The choice of anticoagulant in blood collection tubes is also important, as citrate and heparin can affect IL-6 and TNF-α levels, whereas EDTA provides superior stability [180].

Due to these factors, the lack of standardized methods and established reference ranges for cytokines limits their use in routine clinical practice. However, CRP remains a well recognized inflammatory biomarker for assessing inflammation across various conditions. Its production is largely induced by IL-6, which stimulates the transcription of the CRP gene, with IL-6 itself being upregulated by IL-1 and TNF-α. CRP levels also correlate with other cytokines, such as follistatin-like 1 (FSTL-1) and apelin-13 (AP-13) [181,182]. Consequently, CRP is often used as a surrogate marker for cytokine activity. Elevated CRP, typically measured with high-sensitivity assays (hsCRP), is linked to poor prognosis in HF patients, with an increased mortality risk 12 months after discharge, independent of other CV risk factors [183,184]. CRP levels are also associated with inflammatory coronary events, as patients with elevated CRP have higher rates of HF hospitalization or death post-STEMI [185].

Thus, CRP has become the principal biomarker for assessing inflammatory burden in clinical settings due to standardized testing, a relatively long half-life, and extensive prognostic data.

### 4.3. The Role of Cytokines in Physical Activity

Physical training, particularly in professional athletes, induces a range of structural and biochemical changes, with the immune system and inflammatory response playing key roles in this physiological adaptation. As mentioned earlier, muscle contraction triggers the release of various cytokines, notably myokines such as myostatin, irisin, mitsugumin 53, meteorin-like, apelin, and FSTL-1. Some of these myokines are associated with CVD such as AMI, HF, diabetes, and valvular disease [161]. Additionally, IL-6, IL-8, vascular endothelial growth factor (VEGF), and monocyte chemoattractant protein-1 (MCP-1) significantly increase post-exercise and serve as markers of CV demand and cardiorespiratory fitness [6].

However, immune-inflammatory markers vary between sports, with lower CV-demanding sports often associated with higher levels of pro-inflammatory cytokines compared to high-demand sports [186]. Among cytokines, IL-6 is the most widely studied in relation to exercise, as it is abundantly produced and released by muscle cells [178]. IL-6 plays a critical role in regulating the balance between Th1-mediated pro-inflammatory and Th2-mediated anti-inflammatory responses, with levels rising exponentially with the duration and intensity of exercise [178]. For instance, plasma IL-6 levels may increase 50- to 100-fold during ultra-endurance events [187]. Interestingly, IL-6, along with TNF-α and CRP, tends to decrease at rest following a training program. Notably, coronary artery disease patients in cardiac rehabilitation programs with aerobic exercise show reduced levels of circulating pro-inflammatory cytokines, including IL-6 [188].

Cytokine patterns differ depending on the type and intensity of exercise. For example, interferon-gamma (IFN-γ) and TNF-α levels increase rapidly after exercise and decline quickly, whereas IL-1β and IL-6 levels rise more gradually and remain elevated for several hours [189]. IL-18, on the other hand, decreases after aerobic exercise [190]. Cytokines like TNF-α and IL-1β are more responsive to exercise intensity, while IL-8 and IL-17 are associated with prolonged exercise duration [191]. Moreover, IL-2, which promotes T lymphocyte differentiation and suppresses inflammatory responses, shows a unique secretion pattern that correlates with the regularity and intensity of exercise [192].

IL-10 is another key anti-inflammatory cytokine. Heavy training loads are associated with increased numbers of Th2 and regulatory T-cells, which produce IL-4 and IL-10, shifting the immune system towards an anti-inflammatory state. IL-10 levels increase post-exercise in proportion to the exercise duration [193].

Additionally, IFN-γ, part of the interferon cytokine group, decreases after moderate and strenuous exercise but rises with consistent moderate exercise over a month. This response likely serves to limit inflammation and prevent tissue damage [194].

Chemokines, which guide immune cell movement, also play a role in exercise-induced immune responses. CXCL-8, produced by muscle cells, increases rapidly after various exercise types, especially intensive ones [195,196]. Other chemokines, such as monocyte chemotactic protein-1 (MCP-1) and macrophage inflammatory protein (MIP), peak after exercise as well [196,197].

In patients with type 2 diabetes and macrovascular atherosclerosis, higher circulating IL-6 levels are observed compared to those with atherosclerosis alone. Combined with TNF-α levels, IL-6 levels may improve the prediction of atherosclerosis development in these patients [198]. TNF-α is closely linked to insulin resistance, obesity, and metabolic diseases. Exercise-induced IL-6, in turn, stimulates the production of anti-inflammatory cytokines like IL-10 and IL-1ra while inhibiting TNF-α release, lowering its levels in the bloodstream. IL-1ra production also increases during exercise, returning to baseline within 24 h [199]. These shifts in inflammatory state underscore the protective role of exercise against CVD and diabetes mellitus [200].

IL-15, another myokine, influences adipogenesis by reducing adipocyte proliferation, altering adipocyte size, and promoting apoptosis. A meta-analysis found higher IL-15 levels post-exercise, particularly after resistance exercises compared to aerobic ones [201]. IL-13 is also involved in exercise-related metabolic changes, helping to preserve glycogen stores and enhance fatty acid oxidation and mitochondrial respiration. These mechanisms support prolonged energy supply and contribute to favorable glycemic effects [202]. CRP, another inflammatory marker, also correlates with exercise intensity, with higher levels observed following intense exercise.

Cytokine production during exercise depends on factors such as age, sex, exercise intensity, duration, and training level. While exercise-induced cytokines have established anti-inflammatory effects, high-intensity exercise, particularly with insufficient recovery, may dysregulate the immune system, resulting in a pro-inflammatory state and increased illness susceptibility [191]. Key cytokines and their roles in various CV disorders are detailed in Table 1.

### 4.4. Utility of Cytokines as Biomarkers for Athlete Health

Identifying new biomarkers, particularly in elite and professional athletes, is essential for evaluating the effectiveness of training programs, tailoring them to individual needs, and monitoring progress over time. Cytokines, given their role in physical activity, hold significant potential as biomarkers in this context. Elevated cytokine levels, if consistently above normal, could signal a persistent inflammatory state resulting from infection or tissue damage. For example, athletes often sustain various injuries affecting muscles, joints, and bones; persistently high levels of inflammatory markers such as IL-1β, TNF-α, or IL-6 may indicate incomplete healing from previous injuries. High-intensity sports are associated with increased immune-inflammatory and oxidative stress markers, which correlate with greater oxidative stress and a reduced anti-inflammatory profile, potentially lowering tissue healing capacity and prolonging inflammation [186].

In these scenarios, cytokines could serve as valuable biomarkers. However, as mentioned previously, assessing cytokine levels is challenging due to individual variability, differences in exercise types, and fluctuations in the same athlete over time. The most accurate use of cytokines as biomarkers may involve an initial calibration period for each athlete to establish baseline levels [203].

Monitoring serum cytokine levels could be particularly beneficial for diagnosing unexplained underperformance syndrome (UPS), formerly known as overtraining syndrome (OTS). This condition is characterized by a persistent decline in athletic performance despite at least two weeks of relative rest. The underlying mechanisms of UPS are not fully understood, but evidence suggests it may stem from excessive cytokine production following exercise, leading to a pro-inflammatory state. A proposed model implicates increased production or intolerance to IL-6 as a potential cause [204]. As previously mentioned, prolonged exercise triggers an increase in anti-inflammatory cytokines (e.g., IL-10, IL-1ra), potentially impairing the immune system’s ability to mount an adequate response to pathogens and increasing susceptibility to infections and viral reactivation [205]. Indeed, UPS was associated with a higher incidence of upper respiratory tract infections. It was suggested that intense exercise may elevate cytokine production due to tissue trauma and increased circulating stress hormones such as cortisol, catecholamines, and prostaglandin E2, promoting a shift towards a TH2 lymphocyte profile and suppressing cell-mediated immunity, which in turn raises the risk of infections in athletes [206].

Cytokines have the potential to serve as biomarkers for various conditions in athletes, providing valuable insights into training program effectiveness, progress, inflammation status, and optimal recovery periods. As highlighted, cytokines are also important biomarkers in CV disease, which means they could be used to assess cardiorespiratory fitness in athletes or customize training programs to align with CV health. However, as with clinical applications, their use outside structured research protocols is currently limited by technical and logistical constraints, underscoring the need for further research to enable their integration into routine practice.

## 5. Other Emerging Biomarkers and Future Directions

### 5.1. Other Emerging Biomarkers

Emerging evidence indicates that serum levels of the sarcomere protein, cardiac myosin binding protein C (cMyBP-C) and its fragments may, in addition to cTnI, provide a biomarker for severe cardiac stress and MI [207,208,209]. The comparison of cTnT and cMyBP-C variability and detection in humans revealed acceptable parameters of variability [210]. cMyBPC is a critical regulatory protein that interacts with multiple other key regulatory proteins including titin, myosin cross-bridges, and actin-tropomyosin in the thin filament [211]. The versatility of these interactions is important in the control of cross-bridge kinetics, velocity of shortening, the Frank–Starling mechanism, and the activation of the thin filament. Prominent control of these interactions occurs with beta-adrenergic stimulation and phosphorylation at multiple sites on cMyBP-C and with oxidative stress inducing S-glutathionylation (S-Glu-MyBP-C) residues. Experiments reported by Solaro and colleagues were the first to report that the induction of S-Glu-cMyBP-C occurs with oxidative stress in a mouse model of HFpEF [212]. Sarcomeric levels of S-Glu-MyBP-C correlated with slowing of cross-bridge kinetics and diastolic dysfunction. Studies in other labs confirmed and extended these experimental findings, reporting that phosphorylation and S-glutathionylation of cMyBP-C are mutually exclusive [213]. Moreover, increased levels of cardiac S-Glu-cMyBP-C were reported to occur with high stress exercise that increased oxidized glutathione and suppressed phosphorylation by adrenergic stimulation [213]. Increased levels of S-Glu MyBP-C were also demonstrated in human end-stage heart failure [213].

Previous evidence of the release of cMyBP-C into serum with cardiac stress together with evidence of the generation of S-Glu-cMyBP-C in the heart encouraged studies aimed at detecting its serum levels in conditions of oxidative stress in animal models and humans [214]. As indicated above, inflammation and oxidative stress occur in elite athletes in different sports and potential biomarkers were proposed [186]. Oxidative stress associated with cTnT release into serum was identified to occur with acute and chronic exhaustive exercise in a rat model forced to swim with attached weights [215,216]. To assess serum levels of S-Glu-MyBP-C, antibodies were developed to permit immune precipitation followed by analytical determinations [186]. Increased levels were detected in mouse and monkey models of metabolic syndrome/oxidative stress and diastolic dysfunction as well as in a cohort of humans with diastolic dysfunction. Even though these findings on serum S-Glu-cMyBP-C need to be validated by other laboratories and in larger cohorts of patients, the data indicate a need for further experiments determining temporal levels of serum S-Glu-cMyBP-C in humans not only in cardiac stress but during acute and chronic exercise.

Some trophic factors, particularly brain-derived neurotrophic factor (BDNF), also have the potential for translation into biomarkers. Proper cardiac development is known to depend on BDNF/TrkB signaling. Additionally, improved cardiac function and cardiomyocyte survival are associated with higher early post-AMI BDNF. According to a recent in vivo study conducted on the hearts of WT mice exposed to myocardial infarction, BDNF levels increase within 24 h of MI and then fall at four weeks as LV dysfunction, adrenergic denervation, and impaired angiogenesis take place [217]. The potential role of these and other trophic factors to become biomarkers of cardiovascular illnesses requires more research.

### 5.2. Future Directions

The potential role of inflammatory cytokines as diagnostic markers for cancer and CV diseases has been recognized. Although significant progress has been made in understanding cytokines’ roles, establishing clear relationships between cytokine expression and disease progression, survival, and therapeutic response remains a major challenge [218].

Due to their stability and unique properties, exosomes hold promise for future disease treatment. Compared to traditional vectors for gene and drug therapy, such as viruses, nanoparticles, and liposomes, exosomes offer advantages in therapeutic efficacy, the ability to cross biological barriers, precision, immune compatibility, and safety. Thus, advancing our understanding of the biology of these extracellular vesicles is essential to address the technical and practical challenges that currently limit their clinical application [66]. Future developments in exosome therapy will likely emphasize personalized treatment strategies, enhancing therapeutic outcomes and minimizing unnecessary side effects [118].

In terms of technological advancements, the detection of various forms of cTn I in serum represents an expanding field of investigation [60]. Technological progress has improved the ability to identify cTn markers and stratify patients with AMI and coronary artery disease, particularly those experiencing metabolic stress impacting myocyte integrity. However, the interpretation of elevated serum cTn levels—measured using high-sensitivity antibodies in apparently healthy individuals—highlights the need to integrate the detailed knowledge of cTn biology with its clinical appearance in serum [219].

## 6. Conclusions

While troponin elevation indicates myocardial injury, it does not reveal the specific mechanism involved; thus, non-ischemic causes of troponin elevation should be considered when using these advanced assays. This can make differential diagnosis more challenging for clinicians. New assays now enable the detection of elevated troponin levels even in healthy individuals, and these elevations may be associated with future CV issues. Athletes, in particular, require careful assessment, as it is well-documented that intense physical activity can lead to transient troponin elevation. While troponin release after exercise may be attributed to factors such as increased membrane permeability, apoptosis, and transient ischemia, the exact mechanisms remain unclear. The complex biology of troponin, along with the sensitivity of cardiac myocytes to the microenvironment, may explain troponin release during both physiological events like exercise and pathological conditions. Emerging research fields are exploring the roles of cytokines and exosomes in the development, progression, and diagnosis of various CV diseases, as well as in response to exercise. Due to their stability and packaging abilities, exosomes have significant potential in diagnostic, prognostic, and therapeutic applications for complex diseases. Similarly, the use of cytokines as biomarkers shows promise for refining training protocols, predicting CV risks, and improving overall health outcomes. Large-scale studies are needed to examine their roles in specific CV conditions and to evaluate their practical applications in clinical settings.

## Figures and Tables

**Table 1:** Cytokines involved in different cardiovascular conditions and their level changes during exercise.

Cytokine	Associated Cardiovascular Conditions	Level Variation During Exercise
**IL-1 α and β**	HF, Atherosclerosis, CCS, AMI, Arrhythmias, Myocarditis	Increases
**IL-1ra**	Atherosclerosis	Increases
**IL-4**	AMI	Increases
**IL-6**	HF, CCS, AMI, Atherosclerosis, Unstable Angina, Arrhythmias	Increases
**TNF-α**	HF, AMI	Depends on the intensity of exercise; decreases in trained individuals
**IFN-γ**	AMI, HF	Decreases after moderate and strenuous exercise; increases in trained individuals
**IL-17**	Arrhythmias	Increases
**IL-18**	Atherosclerosis, HF	Decreases
**CXCL-8 (IL-8)**	HF, Myocarditis	Increases
**Myostatin**	CCS, Atherosclerosis	Increases

IL, interleukin; TNF, tumor necrosis factor; IFN, interferon; HF, heart failure; CCS, chronic coronary syndrome; AMI, acute myocardial infarction.

## Data Availability

Not applicable.
